# Evaluation of quality of TB control services by private health care providers in Plateau state, Nigeria; 2012

**DOI:** 10.11604/pamj.2014.17.77.3412

**Published:** 2014-01-31

**Authors:** Luka Mangveep Ibrahim, Obinna O Oleribe, Patrick Nguku, Gabriel Chukwak Tongwong, Lakda Gonen Mato, Musa Istifanus Longkyer, Samuel Ogiri, Peter Nsubuga

**Affiliations:** 1 Nigeria Field Epidemiology and Laboratory Training Programme, Nigeria; 2 E&F Management Consult, Abuja, Nigeria; 3 TB and Leprosy control program plateau state, Nigeria; 4 World Health Organization, Nigeria; 5 Global Public Health Solutions, Decatur GA USA

**Keywords:** Private facilities, Tuberculosis, monitoring and evaluation, Logframe approach

## Abstract

**Introduction:**

Tuberculosis (TB) is public health concern in Nigeria. The country uses the Directly Observed Treatment Short course (DOTS) strategy for its control. Plateau state started using the DOTS strategy in 2001 and had the Private health facilities (PHF) as an important stakeholder. We evaluated their contributions to case finding and quality of the services to identify gaps in monitoring and evaluation in the TB control services within the PHF to plan for intervention so as to meet the set target for TB control in the state.

**Methods:**

We used the logical framework approach to identify and analyze the problem. We drew up an objective tree and from the objective tree developed a logical framework matrix including evaluation plan. We also conducted desk review to extract data on case findings, case management and outcomes of the treatment. We interviewed TB focal persons and laboratory personnel using structured questionnaire. The data was analyzed using excel spread sheet.

**Results:**

Of the 127 health facilities with TB patients on treatment 27 (21.3%) were PHF. The PHF reported 54.6% (1494) of TB cases in 2011. The sputum conversion rates, cured rate, treatment success rate, and default rates were 85%, 73%, 81.4% and 6.6% respectively. The discordant rates were 3.1% and 1.2% for the state and private health facilities respectively.

**Conclusion:**

Log frame approach is a useful tool for evaluation of TB control services and helps provide evidence for decision making to improve quality of the TB services in the public and private health facilities in the state.

## Introduction

Tuberculosis (TB) still poses major public health problem in the world almost 2 decades after it was declared a global emergency by World Health Organization (WHO). The disease is a public health concern in Nigeria as in other developing countries of the world. According to WHO report of 2011, the country had an estimated incidence and prevalence of 133/100,000 and 199/100,000 population respectively placing it among the top 10 high TB burden countries in the world [1]. Nigeria adopted the WHO Directly Observed Treatment Short course (DOTS) strategy to control the disease using the 8 months treatment regimen in 1996. The key determinants of the quality of the TB control program services under the DOTS strategy include: a) the proportion of patients whose sputum converted to smear negative after the intensive phase (first 2 to 3 months) of treatment among all patients whose sputum tested positive for acid fast bacilli (AFB) at diagnosis and registration for treatment. b) The outcomes of the treatment at the end of 8 months and c) the proportion of slides with discordant results from among all slides tested for quality assurance. Discordance occurs when there is difference in the sputum smear result obtained from the laboratory where the patient was first tested and the result obtained from the same slide by the first level quality controller necessitating rechecking by a second controller (usually a senior officer) who serves as a tie breaker. The targets set by the National TB control program in line with the WHO and Stop TB Strategy are a) treatment success rate of at least 85%, b) default rate of less than 3% and c) the discordant rate of not more than 0.5% among all slides that are examined for quality assurance [2]. The treatment success and default rates are proxy indicators of the quality of TB patient management by the health care workers while the discordant rates indicate the quality of the performance of laboratory personnel in sputum microscopy examination. The laboratory is considered the backbone of the TB control program because no diagnosis of TB is complete without laboratory confirmation. It is the laboratory that determines which patients is cured from the disease and which patient is failing the treatment.

The provision of TB control service takes place in both private and public health facilities in Nigeria as in other parts of the world. Private health providers are important stakeholders in the TB control program in Nigeria. The WHO, in recognition of their significant role in the TB control services, introduced the “Public-Private Mix for TB care” this is a concept that enables participation of all health care providers including the Private health providers in TB control services. Engaging all providers including the private health facilities has been incorporated as the fourth component of the WHOs’ new Stop TB strategy [3].

Plateau state in Central Nigeria had an estimated population of 3.8 million people in 2012 from the 2006 National census. It is an agrarian society and a large segment of the population visit private health facilities for health care. In 2008, of the 964 registered health facilities in the state, 466 (48%) were private health facilities [4]. They have been recognized as important partners in the provision of TB control services in the state and three private health facilities were included among the five pioneering TB diagnosis and treatment sites at the inception of DOTS in 2001. The state TB control program embarked on expansion and decentralization of the TB control services in both public and private health care such that in 2012, there were 38 diagnostic and 127 centers with patients on treatment [5]. The aim of the expansion and decentralization was to ensure accessibility of the services to the patients. However, despite these efforts the state is yet to reach the at least 85% treatment success rate target for TB control. Furthermore, although TB case notification and the outcomes of TB treatment are evaluated quarterly for the state, the actual individual contribution of the public and private health care providers to the case detection in the state is not known. Similarly, the quality of the TB control services in terms of patient management and performance of laboratory in sputum examination in the private health facilities had not been evaluated. The non availability of this vital information affects the state TB control program's capacity to direct intervention to improve the case finding and quality of the TB control services in the state.

We performed an evaluation to determine the contribution of private health care facilities to TB case finding, to assess the availability of trained personnel, and the quality of the TB control services, and to identify monitoring and evaluation gaps in TB control services in the private health care facilities so as to make evidence based decisions and plan to achieve the target for TB control in the state.

## Methods

### Evaluation setting

**Figure 1:**
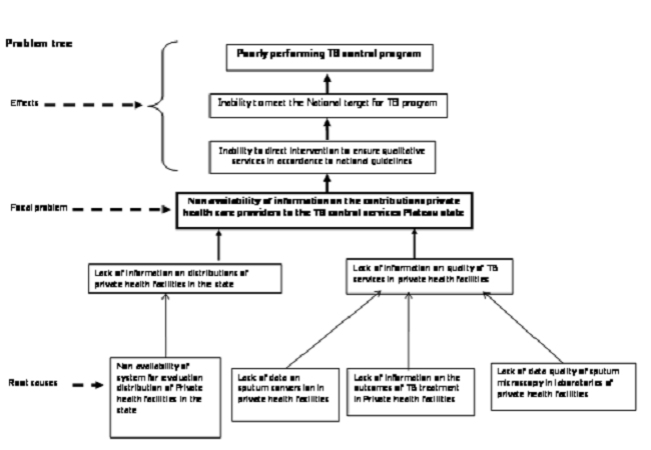
Problem tree evaluation of quality of TB services in Private health facilities in Plateau state 2012

**Figure 2:**
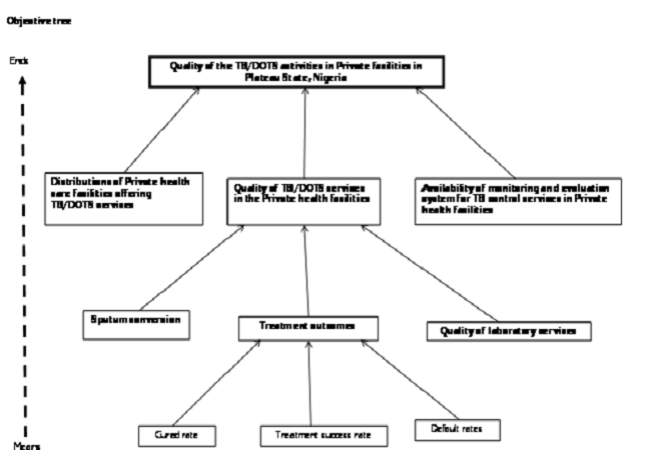
Objective tree for evaluation of quality of TB services in Private health facilities in Plateau state 2012

This evaluation was conducted in August 2012 in Plateau state. It is one of the 36 states in Nigeria located in the North central geopolitical zone. The evaluation began with constitution of an evaluation team made up of an Epidemiologist, a Monitoring and Evaluation Officer, and two states and one Local Government Area (LGA) TB supervisors. We used the logical framework approach (LFA) to identify and analyze the problem (Figure 1). The LFA is both an analytical and management tool used by managers of projects or programs to plan and to monitor their project or program during implementation. The tool also makes provision for the application of the views of all the relevant stakeholders in the project or program. The LFA also helps planners and evaluators to think and to identify the local linkages between the set of means to a set of ends in the project or program [6–7]. We drew up an objective tree (Figure 2) and from the objective tree we developed a logical framework matrix and an evaluation plan (Table 1).

**Table 1:** The Log frame Matrix

	Intervention logic	Objectively Verifiable indicators	Means of verification	Assumptions/Risks
**Overall objectives**	1.1.1. To contribute to achieving the national target at least 85% treatment success	2.1.1. Treatment success rate of cohort of TB patients by end of twelve months	3.1.1. State TB quarterly reports	
**Project purpose**	1.2.1. To ensure services in the private health facilities meet the needs of the TB patients	2.2.1. Defaulters from TB treatment reduce to less than 3% 2.2.2: Treatment success increase to at least 85% 2.2.3: Discordant slides reduced to less than 0.5%	3.2.1. Quarterly state TB reports	4.2.1. There is industrial harmony in state.
**Results**	1.3.1. Adequately trained personnel available for TB control services in Private health facilities	2.3.1. Proportion of Private health facilities with at least one trained personnel for TB control service in next six months	3.3.1. Quarterly LGA TB reports	4.3.1. Staff not be transferred after training
	1.3.2. Adequately trained staff to do sputum smear microscopy in private health facilities	2.3.2. Proportion of laboratories with at least one trained staff on sputum microscopy by end of six months	3.3.2. Quarterly LGA TB reports	4.3.2. Staff not be transferred after training
	1.3.4. Patients adhere to TB treatment	2.3.4. Proportion of patients who complete treatment at end of eight months	3.3.4. State TB quarterly report	4.3.4. Availability of staff
**Activities**	1.4.1. Train staff in private health facilities on TB/DOTS services	2.4.1. Number of training conducted out of trainings planned in a quarter	3.4.1. Quarterly training reports from the state	4.4.1. State government release fund
	1.4.2. Train Lab. staff on sputum microscopy	2.4.2. Number of training conducted out of trainings planned in a quarter	3.4.2. Quarterly training reports from the state	4.4.2. State government release fund
	1.4.3 Provides SOPs for laboratories in PHF	2.4.3: Numbers of laboratories with SOPs	3.4.3: Quarterly supervision reports	4.4.3 SOPs is available
	1.4.4. Offer supportive supervision on laboratory staff by quality control officer	2.4.4. Number of supervisory visits conducted out of planned visits in a quarter	3.4.4. Quarterly QAO supervision reports	4.4.4. Fund is available
	1.4.5. Educate patients on TB disease and adherence to treatment	2.4.5. Proportion of patients with knowledge of TB, duration of treatment and consequences of defaulting treatment	3.4.5. Quarterly LGA supervision reports	4.4.5. Availability of trained health care workers
	1.4.6. Provide Laboratory consumables available for sputum test	2.4.6. Proportion of laboratories offering TB services reached with recording and reporting materials in a quarter	3.4.6. Quarterly LGA reports	4.4.6. Regular supply of material from central unit
Preconditions	Plateau state government will maintain her political commitment to the TB control program GFATM will maintain their supports for the TB control in the state			

### Definition of terms

We used the WHO classification for evaluation of patients after treatment as follows: Cured: is a patient who was initially smear positive at start of treatment, has completed the full course of treatment and has remain smear negative at the end of the seventh month and at least in one other occasion; Treatment completed: defined as patient who completes the full course of anti-TB treatment but does not meet the condition of cured. This definition applies to smear positive, smear negative and extra-pulmonary TB patients; Treatment success: is the combination of cured and completed treatment; Failure: is patients who still remain sputum smear positive at end of fifth or seventh month of treatment; Defaulted: applies to a patient whose treatment was interrupted for at least 2 months; Died: these are patients who died for any reason during the course of TB treatment; Transferred out: refers to a patient who has been transferred to another treatment center in another state and whose treatment result is not known; Discordant slide: A slide is discordant when the results differed between what the staff in laboratory reported and what the expert review found. This is an exercise that is conducted every quarter in the TB control program; slides are sampled from each laboratory for quality check by experts.

### Data collection and analysis

We used a mixed method approach in this evaluation comprising of qualitative methods which were desk reviews and quantitative methods with structured questionnaires. We used predesigned checklist, we extracted information from the state TB reports on distribution of private health facilities participating in TB control services in the state, the contributions of private health facilities in TB case findings, proportions of patients whose sputum test showed negative results at the end of the intensive phase of treatment, and outcomes of the treatment after the 8 months in both private and public health facilities. We also reviewed the reports of Quality Assurance Officer (QAO) of the sputum smear results from the laboratories using a checklist. We used the findings of the desk review on the distribution of private health facilities in the state as guide to identify and interviewed TB focal persons with pre-designed structured questionnaire. We also interviewed laboratory personnel that were participating in sputum microscopy. In an LGA with more than one private health facilities that were eligible for selection, a facility was selected using simple random sampling technique. Data were entered in a Microsoft Excel spreadsheet and descriptive analysis was done.

### Ethical consideration

Permission for the evaluation was obtained from the state ministry of health. We obtained informed consent from all individuals who participated in the study.

## Results

### Distribution and contribution of private health facilities in TB case notification

Of the 127 centers with TB patients on treatment in the state 27 (21.3%) were private health facilities spread across 11 (64.7%) of the 17 Local Government Areas, 15 (55.6%) of the private health facilities were owned by religious organizations (i.e, private for non -profit facilities). Furthermore, 1494 (54.6%) of the 2738 TB patients enrolled for treatment in the state in 2011were by the private health facilities (Table 2). Of the 26 TB focal persons interviewed, 19 (73.1%) had attended workshops on TB control services and only two (7.7%) had not received any form of training (Table 3), furthermore, of the 22 laboratory personnel involved in TB control services in private health facilities, 18 (81.8%) had attended workshops on TB smear microscopy and reporting.

**Table 2:** Contributions and quality of TB services in Private health facilities Plateau state, 2012

Parameter studied		Total in the state	Public (%)	Private (%)
**Case findings**	Total patients enrolled	2738	1244 (45.4)	1494 (54.6)
	Total smear positive patients	957	483 (50.5)	474 (49.5)
	Total counseled for HIV	2317	931 (48.2)	1386 (59.8)
	Total tested for HIV	2038	805 (39.5)	1233 (60.5)
	Total HIV positive	1001	403 (40.3)	598 (59.7)
**Quality assurance reports**	Total diagnostic centers	36	22 (61)	14 (39)
	Total sputum (slides) examined	17,293	7985 (46.2)	9,308 (53.8)
	Total smear positive recorded	2,105	882 (41.9)	1,223 (58.1)
	Total slides evaluated for quality	1438	839 (58.4)	599 (41.6)
	Total rates	33 (2.3%)	26 (3.1)	7 (1.1)
**Sputum conversion**	Number enrolled	999	516 (51.7)	483 (48.3)
	Converted	754	340 (65.9)	414 (85.7)
	Sputum positive	29	15 (2.9)	14 (3.4)
	Continuing treatment	179	164 (31.8)	15 (3.6)
	Died	17	10 (1.9)	7 (1.7)
	Defaulted	14	10 (1.9)	4 (1.0)
	Transfer out	13	6 (1.2)	7 (1.7)
**Treatment outcome**	Number enrolled in 2011	2980	1706 (57.2)	1274 (42.8)
	Treatment success	2371	1334 (78.2)	1037 (81.4)
	Defaulted	221	137 (8.0)	84 (6.6)
	Died	218	121 (7.1)	97 (7.6)
	Treatment failure	18	9 (0.5)	9 (0.7)
	Transferred out	79	32 (1.9)	47 (3.7)

**Table 3:** Availability of trained personnel for TB control services in Private health facilities in Plateau state 2012

Parameter studied		Frequency	Percentage
**Training received by TB focal persons**	No training	2	7.7%
	On-the-job training	5	19.2%
	Workshop	19	73.1%
**Training received by lab. personnel**	On-the-job training	5	20.0%
	Workshop	20	80.0%

### Quality of TB control services

The sputum conversion rate at the end of the intensive phase (i.e, 2 months and 3 months of TB treatment for category 1 and category 2 respectively) was 84.5% in the private health facilities and 75.5% in the state (Table 2). The outcomes of treatment of patients registered in 2010 revealed that treatment success, default and death rates were 81.1%, 7.0% and 7.5% respectively in the state. The treatment success, default and death rates were 82.7%, 4.4% and 7.6% respectively in the private health care facilities (Table 2).

A total of 1438 slides were evaluated for quality assurance, of which 599 (42%) were from private health facilities. The proportion of slides with discordant results (i.e., where the results differed between what the laboratory personnel found and what the expert review found) were 33 (3.1%) for the entire state and 7 (1.1%) in private health facilities (Table 2).

## Discussion

We found in this evaluation that the private health facilities are making significant contributions to the TB control services in Plateau state. The evaluation results also revealed that the quality of the TB services in the private health care facilities compared favorably with those of the entire state. We also demonstrated that we could use the LFA in addressing an important public health evaluation.

Accessibility of services to patients and satisfaction with the services (including the quality of the patient/provider relationship) is among the several factors that motivate TB patients, to patronize any health service provider and adhere to the treatment [8]. The private health facilities are known to be widely spread even to remote areas making services more accessible to patients. Although the private health facilities in our study constituted only 21% of all the facilities providing TB services in the state, they registered more than 50% of the cases indicating there could be some hidden reasons attracting the patients to the private sector. We can opine that these reasons may include relationship, accessibility of the services, privacy/confidentiality, and quality of care and attitudes of the health care workers leading to better providers-patients relationship which are key determinants of client satisfaction [9–10].

Our results showed that majority of the private health facilities were owned by religious organizations which could serve as additional reasons for the high patronage by patients who might enjoy the empathy and perceived better care in those facilities. The preference of private health facilities by patients has been reported by Hazarika in a population-based study in India. The researcher noted that patients’ preference to the private health facilities was due mainly to their dissatisfaction with the services in the public health facilities [11]. Furthermore, the quality of the services in the private health facilities could also be a major drive for the higher patronage by the patients. Our results showed that private health facilities produced good results: the sputum conversion and treatment success rates were higher in the private facilities compared to public facilities, furthermore, the lower default and discordant rates recorded in the PHF compared to public health facilities are indicators of better quality of patient management in the private health facilities. It might have been in this light that the WHO put forth the concept of public private mix to ensure access to high-quality diagnosis and patient care [3]. This point is buttressed by the findings of Lönnroth et al in Ho Chi Minh City of Vietnam. They showed that private health care facilities do play important role in TB because considerable proportions of them have contacts with a private service providers [8]. For effective service and to meet the needs of the patients the services must be taken to the patient seek care.

Training of staffs who are involved in the provision of TB control services is an important component of TB control program because it is one of the factors that influence how the health care workers manage the TB patients. In the DOTS strategy, the health care worker is in contact with the patient through the duration of treatment. Kaur et al in their study on knowledge and attitude of staff, who provide direct observation of treatment to TB patient in Patial India, reported that knowledge of the staff on sputum was better among trained compared to untrained staff [12]. The result of our study revealed that majority of the TB DOTS focal persons and laboratory personnel had attended relevant workshops to equip them with the required knowledge and skills for quality service provision. We can infer from this finding that there exist well trained staff in staff in the private health facilities for TB control services and this must have contributed to the quality of the care for the patients.

### Usefulness of LFA

The application of this tool highlighted the importance of participation of important stakeholders in evaluation of program. It also helped us (the evaluation team) to identify the problem that needed to be addressed including formulation of objectives for the evaluation. It guided our thoughts and served as a reference document on how to carry out the evaluation including implementing the expected results.

### Limitation to the study

We did not explore the contribution of the informally trained private providers such as the traditional healers and patent medicine vendors who play significant role in provision of health care services especially in the rural areas. This category of service providers are widely patronized by the public.

## Conclusion

The private health facilities are making significant contributions to TB control services in diagnosis and patients management in Plateau state. Their contributions are critical for the success of the TB control program in the state and the country. Establishing a state wide monitoring and evaluation system will further improve the quality of the TB control services in Private health facilities through evidence base decision making. Also, the state TB control program should support the Private health facilities to establish system for regular evaluation of the progress of the TB control services in their facilities. The National TB control program should ensure active participation of the private health care providers in the TB control services and they should be motivated to partake in the monitoring and evaluation of the services within their domain to foster ownership and decision making. The Plateau state and the National TB control program should, in addition, conduct further studies on the drivers for patients’ patronage of the private facilities. The Plateau state Ministry of health should train their personnel on Logical Framework Approach and apply the tool for planning, management and evaluation of public health programs particularly the TB control program. There Plateau state TB control program should conduct further studies on factors that motivate TB patients’ patronage of private health facilities in the state.
